# Substance-induced catatonia: the case of synthetic cannabinoids

**DOI:** 10.1192/j.eurpsy.2026.10312

**Published:** 2026-07-31

**Authors:** A. K. Sikora

**Affiliations:** Ivano-Frankivsk National Medical University, Ivano-Frankivsk , Ukraine

## Abstract

**Abstract:**

Catatonia is a potentially life-threatening psychomotor syndrome that can complicate substance intoxication and may be missed in acute psychiatric and addiction settings. Synthetic cannabinoids (e.g., Spice/K2) are associated with severe neuropsychiatric presentations including agitation, psychosis, autonomic instability, and diagnostic ambiguity. This talk presents a clinical case of a 17-year-old male admitted with first-episode psychosis and behavioural disorganisation after synthetic cannabinoid use (positive K2 toxicology). Despite initial management for acute psychosis, the patient developed catatonic features including elective mutism, rigidity and impaired self-care, accompanied by escalating agitation and hallucinations. The case highlights key diagnostic dilemmas (catatonia vs delirium, intoxication, NMS/serotonin toxicity), practical bedside assessment, and evidence-based management principles, including early consideration of benzodiazepines, medical monitoring, and avoidance of potentially harmful treatment delays. The presentation emphasises that recognition of substance-induced catatonia enables timely intervention and prevention of complications such as dehydration, rhabdomyolysis, aspiration and cardiovascular instability.

**Disclosure of Interest:**

None Declared

